# Mapping Winter Wheat with Multi-Temporal SAR and Optical Images in an Urban Agricultural Region

**DOI:** 10.3390/s17061210

**Published:** 2017-05-25

**Authors:** Tao Zhou, Jianjun Pan, Peiyu Zhang, Shanbao Wei, Tao Han

**Affiliations:** 1 College of Resources and Environmental Sciences, Nanjing Agricultural University, Nanjing 210095, China; xinyangzhoutao@163.com (T.Z.); peiyuzhang163@163.com (P.Z.); njauhantao@163.com (T.H.); 2 College of Public Administration, Nanjing Agricultural University, Nanjing 210095, China; 2015109011@njau.edu.cn

**Keywords:** winter wheat classification, Sentinel-1A, multi-temporal, Landsat-8, urban agricultural region

## Abstract

Winter wheat is the second largest food crop in China. It is important to obtain reliable winter wheat acreage to guarantee the food security for the most populous country in the world. This paper focuses on assessing the feasibility of in-season winter wheat mapping and investigating potential classification improvement by using SAR (Synthetic Aperture Radar) images, optical images, and the integration of both types of data in urban agricultural regions with complex planting structures in Southern China. Both SAR (Sentinel-1A) and optical (Landsat-8) data were acquired, and classification using different combinations of Sentinel-1A-derived information and optical images was performed using a support vector machine (SVM) and a random forest (RF) method. The interference coherence and texture images were obtained and used to assess the effect of adding them to the backscatter intensity images on the classification accuracy. The results showed that the use of four Sentinel-1A images acquired before the jointing period of winter wheat can provide satisfactory winter wheat classification accuracy, with an F1 measure of 87.89%. The combination of SAR and optical images for winter wheat mapping achieved the best F1 measure–up to 98.06%. The SVM was superior to RF in terms of the overall accuracy and the kappa coefficient, and was faster than RF, while the RF classifier was slightly better than SVM in terms of the F1 measure. In addition, the classification accuracy can be effectively improved by adding the texture and coherence images to the backscatter intensity data.

## 1. Introduction

Wheat is one of the world’s major food crops [1]. With the acceleration of urbanization in China, the amount of cultivated land has been decreasing, and food security has become an important issue. Winter wheat classification is the basis for acreage and yield estimates, which are important for public policy makers to develop food policies and economic plans [2,3]. The development of remote sensing technology provides a rich data source for crop classification. Remote sensing has the characteristics of frequent and large area detection, and provides a viable method for wheat classification [1,4,5]. Optical remote sensing satellites have been widely used for crop type classification [6]. However, optical remote sensing is susceptible to cloudy and rainy weather, and it is difficult to obtain ideal optical images in the critical period of winter wheat growth in Southern China. Compared with optical remote sensing, synthetic aperture radar (SAR) has all-weather, day and night imaging, canopy penetration, and high-resolution capabilities [7,8,9]. Due to these advantages, SAR has become an effective source of data for crop classification.

Although the classification of SAR images is more difficult than that of optical images [10], several studies have used SAR data for crop monitoring and mapping [11,12,13]. Shao et al. [14] used multi-temporal RADARSAT-1 data to identify rice in the Zhaoqing area of Guangdong Province, China, with a classification accuracy of 91%. Silva et al. [15] compared the use of VV polarization, HV polarization, and HH polarization for crop classification; the results showed that the classification accuracy of HH polarization was better than VV polarization and HV polarization. McNairn et al. [16] used C-band SAR data to classify wheat, maize, soybean, and other crops, and found that multi-polarization SAR data classification accuracy was higher than that of single-polarization SAR data. Ferrazzoli et al. [17] also suggested that increasing the polarization mode can increase the classification accuracy. Several researchers have also found that, compared with single-frequency SAR data, multi-frequency SAR data can effectively improve the classification accuracy [18,19,20]. 

It has also been demonstrated that the addition of texture and coherence features can improve classification accuracy compared to backscatter intensity images alone in crop classification [20,21,22,23]. Jia et al. [20] reported that the use of texture features can improve the classification accuracy of SAR data. Similar results were reported by Yayusman and Nagasawa [23], who used a gray-level co-occurrence matrix (GLCM) to extract SAR texture features for crop classification and found that texture features were useful for crop classification. In the work of Parihar et al. [21], the classification accuracy was improved by adding coherence information to the backscatter intensity data compared to the use of backscatter intensity data alone. Liesenberg and Gloaguen’s [24] research showed that when the texture and coherence information were added, the overall accuracy was improved. Therefore, this study evaluated the classification accuracy of winter wheat using texture and coherence features. Due to the influence of SAR image speckle effects, resulting in inter-class confusion [25], many researchers have studied the combination of optical and SAR images to improve classification accuracy [26,27]. The classification accuracy of maize, soybean, and sunflower increased by 6.2%, 16.2%, and 25.9%, respectively, when multi-temporal SAR images were added to optical images [26]. Kussul et al. [28] used the RADARSAT-2 and EO-1 to classify Ukrainian crops, and found that the combination of SAR and optical images gave better accuracy. In this paper, the combination of SAR and optical images for improving crop classification accuracy was also evaluated. The selection of classification algorithms directly affects the classification results. Support vector machine (SVM) [29], random forest (RF) [30], and neural network (NN) [31] are the most commonly-used classifiers for SAR data classification. Wang et al. [32] used SAR and optical images to classify land cover and found that the RF algorithm was better than SVM when classifying various land covers using pixel-based image analysis. Other studies have also demonstrated the effectiveness of SVM for SAR data classification [3,33]. 

However, few reported studies have involved crop classification at the early stages of agriculture; particularly there is a lack of use of multi-temporal SAR data for such studies. The use of remote sensing data at the end of the season, or later, to obtain crop acreages is not useful for supporting in-season crop management [34], such as yield forecasting and irrigation management [11]. Some studies have used the combination of SAR and optical images for crop classification. Forkuor et al. [9] studied the contribution of SAR images to crop classification when used as a supplement to high-resolution optical images. Villa et al. [34] combined multi-temporal SAR and optical data for in-season crop mapping. However, they used the combination of SAR and optical images to classify and did not investigate the ability of SAR data alone for its use in in-season crop mapping.

The above studies demonstrated that SAR data produced good classification accuracy. However, SAR data have rarely been studied in the classification of winter wheat in urban agriculture regions with complex planting structures and fragmental planting parcels, which are more difficult to classify than those with simple planting structures or less fragmented parcels. Particularly, there is a lack of such studies using multi-temporal Sentinel-1A images for in-season winter wheat mapping. The purpose of this paper was to explore the feasibility of in-season winter wheat mapping and to improve classification accuracy by using SAR data, optical images, and the combination of both types of images to classify winter wheat in an urban agricultural region with a complex planting structure in Southern China. For this objective, interference coherence, texture, and backscatter intensity images were first extracted from SAR data during winter wheat growth. Then, the satisfactory classification results were successfully identified based on these Sentinel-1A-derived information and optical images, and using the RF and SVM classifiers. The results of this study are important not only in the urban agriculture region, but also in other areas of simple planting structures. This study is particularly important for in-season winter wheat mapping of SAR data and compares the effects of the addition of coherence and texture images on winter wheat classification. 

## 2. Study Area and Datasets

### 2.1. Study Area

**Figure 1:**
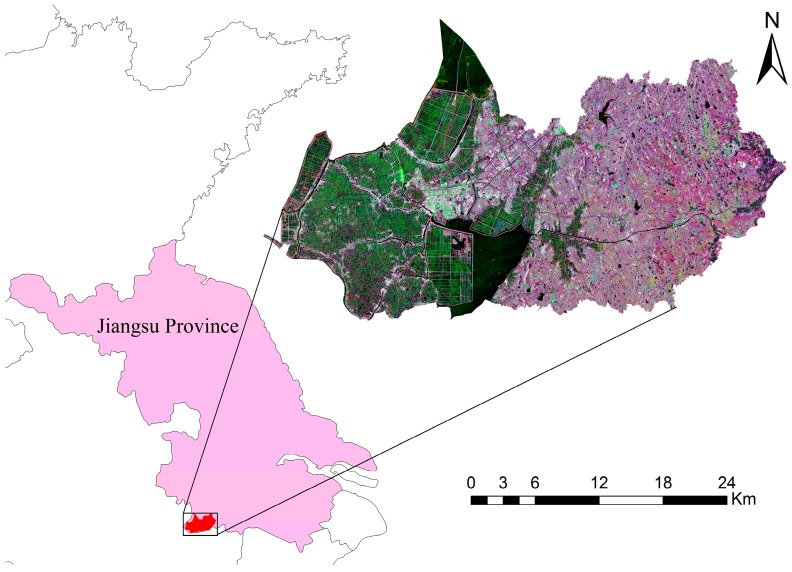
The study area is located in the southwest of Jiangsu Province, China and an overview of the SAR data (R: 2015-11-12 VH polarization, G: 2016-03-21 VV polarization, B: 2016-05-08 VH polarization).

The study site is located in a typical urban agricultural region in Gaochun District of Nanjing, the capital of Jiangsu province, China, with central coordinates of 118°52’ E and 31°19’ N (see Figure 1). The area, covering a total area of 802 km^2^, has a subtropical monsoon climate with an annual average temperature of 16.0 °C and abundant rainfall (the annual average rainfall is 1157 mm). The area has a complex topography, being low in the west and high in the east; the west is a plain, and the east is a low hilly area. The main winter crops are winter wheat and winter rapeseed. The winter wheat growth period is late October to the following June. Winter rapeseed is sown in early October and harvested in mid-May of the following year. Although the study area is not the main crop production area in China, it is a representative urban agricultural region in Southern China due to its abundant rainfall and complex crop planting structure.

### 2.2. SAR Satellite Data

Sentinel-1 is an important component of the Global Monitoring for Environment and Security (GMES) [35], a joint initiative of the European Commission and the European Space Agency (ESA), consisting of two satellites of the constellation. Sentinel-1A was launched on 3 April 2014, while Sentinel-1B was launched on 25 April 2016 [3]. Sentinel-1A provides C-band images in both singular and dual polarization with a 12-day repeat cycle [36], and has four imaging modes: strip map (SM), interferometric wide swath (IW), extra wide swath (EW), and wave (WV), each with different resolutions. In this study, six Sentinel-1A images in IW mode with a dual polarization scheme (single-look complex (SLC) products) were acquired from the ESA (European Space Agency), as shown in Table 1.

### 2.3. Optical Satellite Data

Landsat-8 launched in February 2013 and carries two sensors, the Operational Land Imager (OLI) and the Thermal Infrared Sensor (TIRS). The Operational Land Imager (OLI) is a nine-band push broom scanner with a swath width of 185 km, eight channels at 30 m spatial resolution, and one panchromatic channel at 15 m spatial resolution [37]. Four Landsat-8 OLI images were obtained for the following dates in 2016: 25 February, 12 March, 28 March, and 29 April. These images are Level 1T products downloaded from the USGS (US Geological Survey). In addition, a GaoFen-1 (GF-1) P/MS image with a spatial resolution of 2 m on 29 April 2016 was acquired. As the first satellite of the Chinese High-Resolution Earth Observation System [38], the GF-1 satellite was launched from Jiuquan Satellite Launch Centre (Gansu province, China) in April 2013, and carries two panchromatic/multi-spectral (P/MS) and four wide-field view (WFV) cameras [39]. The acquired GF-1 P/MS (Level 1A product) image served as reference data, with one panchromatic band and four multispectral bands.

### 2.4. Field Survey 

In order to collect land cover information, a field survey was conducted on 17 May 2016. According to the field survey, the land cover types in the study area are winter wheat, winter rapeseed, forest, water body, and urban. The land covers, which are easily identified by visual interpretation, are identified mainly from the GF-1 image, including urban, forest, and water body. GPS coordinates and land cover information for each survey site were recorded and their spatial extent was transferred to a geographic information system (GIS). Fifty percent of the reference data were selected as the training samples by stratified random sampling, and the remaining 50% were used to perform the accuracy assessment (Table 2). All the classifications were conducted at per-pixel basis.

In this paper, a confusion matrix was used to calculate the overall accuracy, and the kappa coefficient and the F1 measure (Equation (1)) were used to evaluate the classification accuracy. The overall accuracy is computed by dividing all correctly-classified pixels by the entire validation dataset [40,41]. The kappa coefficient is computed to determine whether the values in an error matrix are significantly better than the values in a random assignment [3]. Additionally, the F1 measure, as the harmonic mean of the producer’s and user’s accuracy [42,43], is considered to be more meaningful than the kappa coefficient and the overall accuracy [40]. The F1 measure ranges from 0 to 1; a large F1 measure indicates good results, while a small F1 measure indicates poor results.

(1) $$F1\;\mathrm{measure}\;=\;2\times \frac{producer’s\;accuracy\times user’s\;accuracy}{user’s\;accuracy+producer’s\;accuracy}$$

## 3. Methods

### 3.1. Satellite Data Pre-Processing 

In order to reduce errors resulting from instrumental variations in data acquisition, image noise, and misregistration, the optical images were corrected from radiometric and atmospheric effects [44]. The atmospheric correction was done by the ENVI FLAASH model. In this paper, the normalized difference vegetation index (NDVI, Equation (2)) [45] and the simple ratio index (SR, Equation (3)) [46] vegetation index were selected and calculated, and the latter reduced the effect of variable illumination due to topography [47].

(2) $$NDVI=\frac{NIR-RED}{NIR+RED}$$

(3) $$SR=\frac{NIR}{RED}$$

where *NIR* and *RED* correspond to the surface reflectance of band 4 and band 5 of Landat-8, respectively. 

The SAR data were pre-processed by SARscape 5.2 software (Sarmap, Purasca, Switzerland), which included multi-look, registration, speckle filtering, geocoding, and radiometric calibration. A Lee filter with a 3 × 3 window was applied to reduce speckle noise [48]. The SAR data was resampled to a pixel size of 20 m spatial resolution, and the digital number values (DN) were converted to a decibel (dB) scale backscatter coefficient (σ°). Then, the images were geocoded using the shuttle radar topography mission (SRTM) DEM. All data were also geometrically rectified using 30 ground control points, with the root mean square error (RMSE) less than 0.5 pixels. Ground coordinates of these points were provided from the 1:10,000 LULC map developed by Nanjing Institute of Surveying and Geotechnical Investigation.

### 3.2. Feature Sets 

#### 3.2.1. Texture Features

Texture feature is an important feature to improve the classification accuracy of SAR data, which can be quantified by a series of different statistical measures, and is widely used for crop classification [7,20,23,49]. In this paper, the gray level co-occurrence matrix (GLCM) proposed by Haralick, which is widely used in spatial and texture extraction [50], was used to extract texture features. Finally, the following eight texture measures were extracted: mean, variance, entropy, angular second moment, contrast, correlation, dissimilarity, and homogeneity.

#### 3.2.2. Coherence Features

Coherence is a correlation coefficient that explains that small changes in the surface (vegetation, non-vegetation, rock, etc.) occurring during the time interval between two SAR acquisitions [21]. As the coherence size determines whether the corresponding pixel changes, the coherence coefficient ranges from 0 to 1. A large coherence coefficient indicates that the change is small, whereas a small coherence coefficient indicates that the change is large. In this paper, the coherence of the SAR images was calculated using SARscape 5.2 software and 10 coherence images were obtained using the following InSAR data pairs: (1) 22 November 2015 and 9 January 2016; (2) 9 January 2016 and 26 February 2016; (3) 26 February 2016 and 21 March 2016; (4) 21 March 2016 and 14 April 2016; and (5) 14 April 2016 and 8 May 2016.

#### 3.3.3. Feature Combination

In order to explore the ability of SAR variables and optical images to distinguish winter wheat, and to obtain the best combination of features to improve the classification accuracy of winter wheat, the following combinations were considered (Table 3). 

### 3.3. Classifiers 

In this study, two classifiers were used for classification: support vector machine (SVM) and random forest (RF). 

The SVM classifier is a machine learning method based on statistical learning theory [51], which has been widely used in remote sensing data classification and has achieved better classification accuracy [52,53]. It searches the optimal hyperplane by mapping the input vector into the high-dimensional space to separate the training vectors of the two classes into two subspaces [33]. In this paper, the radial basis function (RBF) kernel was implemented, and the kernel parameters kernel width and penalty parameter were set to 0.125 and 100, respectively. 

The random forest (RF) classification algorithm belongs to an ensemble classifier class and is built on multiple decision trees, with each tree being fitted to a different bootstrapped training sample and a randomly-selected set of predictive variables [7,9,32,54]. The final classification or prediction results are obtained by voting [7,54]. A large number of studies have proved that the random forest algorithm has high prediction accuracy [55,56], good tolerance for abnormal values and noise, and is not prone to over-fitting. In this study, 500 trees were generated using the square root of the total number of predictors at each node.

## 4. Results and Discussion 

### 4.1. Analysis of Temporal Variables Used for Classification 

In order to use temporal variables to distinguish winter wheat, the temporal changes of each land cover class were analyzed from the temporal variables derived from optical and SAR images. Then, the separability of the land cover classes was also explored.

#### 4.1.1. Temporal Variables Extracted from SAR Data 

**Figure 2:**
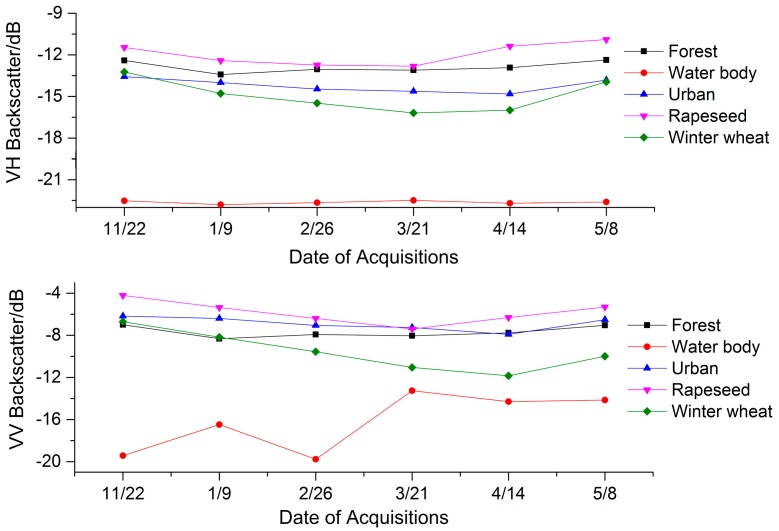
Average backscatter values for each land cover class on six image acquisition dates.

Figure 2 shows the time series of mean backscatter (σ^0^) values for each land cover class. The results show that the temporal variation of the backscatter of different classes is obviously different. The backscatter value of VH polarization compared to VV is relatively small. The average VH backscatter of wheat and rapeseed decreased from −13.2 dB and −11.5 dB on 22 November 2015, to −16.2 dB and −12.8 dB on 21 March 2016, respectively. The average VV backscatter of wheat and rapeseed were attenuated from −6.7 dB and −4.2 dB on 22 November 2015 to −11.9 dB on 14 April 2016 and −7.4 dB on 21 March 2016, respectively. With the growth of wheat and rapeseed, the backscatter values for VV and VH polarization both increased. The VV and VH backscatter of forest were first reduced and then increased to the maximum on 8 May 2016. The results showed that: (1) The VV polarization decay of winter wheat was higher than that of VH, because the wheat has obvious vertical structure by which the VV polarization was affected during the propagation process; (2) In the early stage of growth, soil played a leading role in radar backscatter [57,58]. With the gradual growth of wheat, the leaf density and the rod density gradually increased, and the wheat was more uniformly covered on the ground surface. The dominant position of the surface scattering decreased gradually, and the backscatter value decreased with the increase of the leaf density trend; (3) With the maturation of wheat, the water content of the canopy gradually decreased, so that the scattering effect of the soil gradually increased, which eventually led the VH and VV polarization backscatter to increase [59].

The backscatter values of the water body were very low due to the specular reflection of water, which causes less reflection towards the radar antenna [21,60]. The backscatter values of the urban area were relatively high and remained constant.

**Figure 3:**
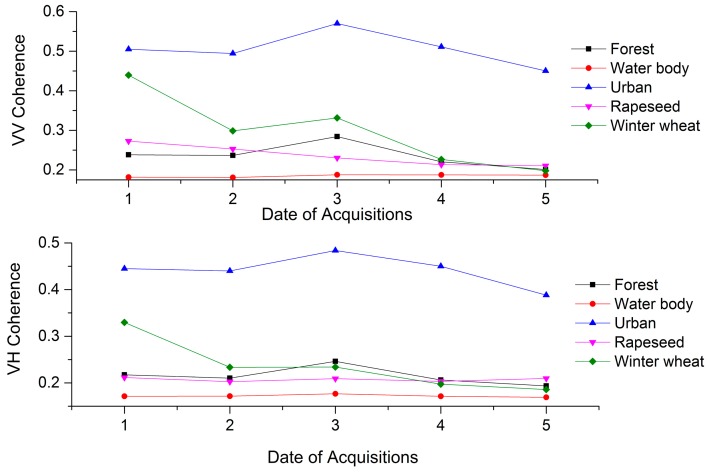
Average coherence values for each land cover class. Note: the meaning of the notations 1, 2, 3, 4, and 5 can be found in Section 3.2.2.

Figure 3 shows the time series of the mean coherence values for each land cover class. The results show that the coherence of the urban land cover was the highest (in the range of 0.45 to 0.57) and the coherence of the water body land cover was the smallest (in the range of 0.18 to 0.19) among these different land covers. Moreover, similar results have been observed in other studies [21]. This is because the water body surface was easily affected by natural factors, such as wind, and was thus extremely unstable, whereas the urban area was rarely affected by environmental factors [61]. Winter wheat had a maximum value of 0.44 generated from the 22 November 2015 and 9 January 2016 InSAR pair; the minimum value of 0.20 was generated from the 14 April 2016 and 8 May 2016 InSAR pair. Blaes and Defourny’s [62] research found that there was a strong correlation between winter wheat height and the coherence value. Thus, the degrees of change of the surfaces were different, and the coherence values of the land covers were different, which provided effective information for the classification of the land.

#### 4.1.2. Temporal Variables Extracted from Optical Data

**Figure 4:**
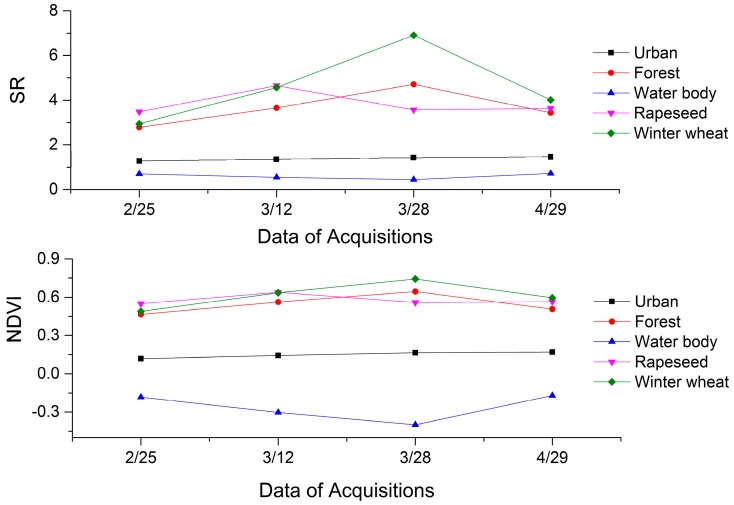
Average SR and NDVI values for each land cover class on four image acquisition dates.

Figure 4 shows the time series of the mean SR and NDVI for each land cover class. As observed in this figure, the SR values of wheat increased first and then decreased (in the range of 3.0 to 4.1), with the highest value of 7.4 occurring on 28 March 2016. The trends of SR and NDVI curves were basically the same; at the end of March there was a peak for winter wheat, and rapeseed had a valley. During this period, rapeseed entered the flowering stage, which contributed to the two vegetation indices’ values decreasing, while after the flowering period the two vegetation indices’ values increased, indicating that winter wheat had the highest separability at the end of March. The vegetation indices’ values over the water body and urban areas were very low. On the other hand, the forest indices’ values were less than that of the corresponding winter wheat. Our results indicate that the time series of the SR and NDVI values of each land cover class were obviously different, which has been widely used in crop remote sensing classification [47].

### 4.2. Winter Wheat Mapping 

#### 4.2.1. SAR Image Classification and Accuracy Assessment 

**Figure 5:**
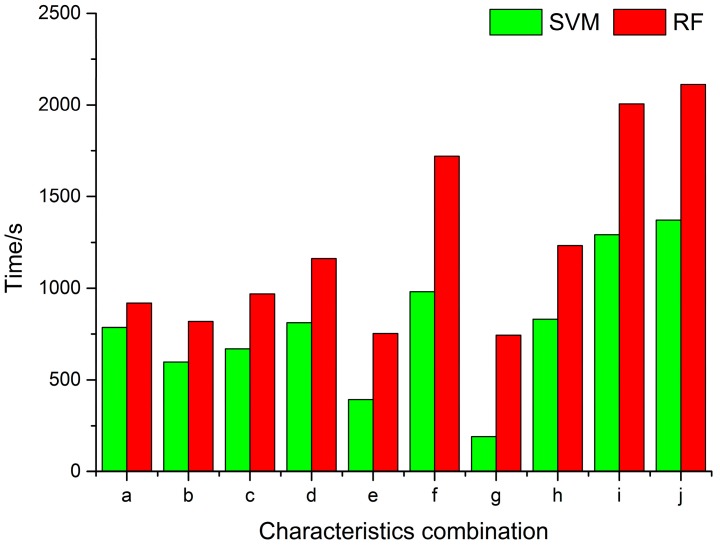
Comparison of processing time of different combinations classification by RF and SVM algorithms.

The classification accuracy of winter wheat using RF and SVM classifiers are shown in Table 4. Compared to the RF using different combinations of SAR data, the results of the SVM were generally slightly higher in terms of overall accuracy and kappa coefficient; moreover, RF cost more time than SVM in all combinations (see Figure 5). However, RF obtained better winter wheat classification results than SVM in terms of the F1 measure. As shown in Table 4, all F1 measures for winter wheat using the single polarization were lower than 75% and the performance of VV polarization was better than that of VH polarization, which indicates that two single polarizations could not meet the winter wheat classification accuracy requirements. The winter wheat classification accuracy using the SAR data combination of VV + VH was much better than that of VH and VV, with an overall accuracy of 91.45% and a kappa coefficient of 0.8729 (F1 measure = 85.72), which was mainly due to winter wheat VV polarization and VH polarization having different scattering mechanisms, and the combined polarization (VV and VH) rather than VV or VH provided richer wheat radar wave scattering information, effectively improving the winter wheat classification accuracy. 

In order to fully explore the Sentinel-1A data to improve the classification accuracy, texture and coherence information were extracted and added to the classification. As shown in Table 4, the classification accuracy using backscatter intensity data was higher than that of the texture and the coherence images. Although the overall accuracy and kappa coefficient of the texture images were higher than that of the coherence images, the F1 measure of the winter wheat was lower than the coherence images. This suggests that backscatter intensity was more important for classification than coherence and texture information. When the coherence images were added to the backscatter intensity images, the overall accuracy increased from 91.45% to 94.94% for RF (F1 measure = 92.35%) and from 89.82% to 96.19% for SVM (F1 measure = 93.10%), respectively. This result was consistent with earlier studies using the combination of backscatter intensity and coherence images [21,22]. Furthermore, when the texture images were added to the backscatter intensity images, the classification accuracy also increased. This indicates that coherence and texture information are useful parameters for the classification of SAR data. Jia et al. [20] also showed that the use of texture images can improve the classification accuracy of SAR data. However, the classification accuracy using the combination of coherence and texture images was lower, with an overall accuracy of 73.82% for RF, and 85.19% for SVM, respectively. 

The best results of winter wheat were obtained using an SAR data combination of VV + VH + C + T, with the overall accuracy and kappa coefficient up to 95.95% and 0.9399 for SVM (F1 measure = 93.61), respectively, which was lower than the F1 measure for RF. In general, when the target accuracy is greater than 85%, the classification of this crop is reliable [26,63,64]. Therefore, only the use of SAR data can meet the winter wheat classification accuracy requirements (with F1 measure than 85%). That is to say, the optical image can be replaced by SAR data in order to distinguish winter wheat with complex planting structures. For the two classifiers, although the F1 measure using VV + VH + C + T was higher than that of VV + VH + C, the overall accuracy using VV + VH + C was better than that of VV + VH + C + T. This indicates that the combination of coherence, texture, and backscatter intensity images can improve the classification accuracy. For forest, urban areas, and water fields, the F1 measure using VV + VH + C + T were better than 90%, with an overall accuracy and kappa coefficient equal to more than 94% and 0.9, respectively. For rapeseed fields, VV + VH + C + T had the best performance of all combinations, while it could not distinguish rapeseed areas well (with an F1 measure of no more than 85%). Compared with the previous research results [26], rapeseed classification accuracy is low because the rapeseed planting structure is complex and the planting area is small. 

#### 4.2.2. Optical Image Classification and Accuracy Assessment 

**Figure 6:**
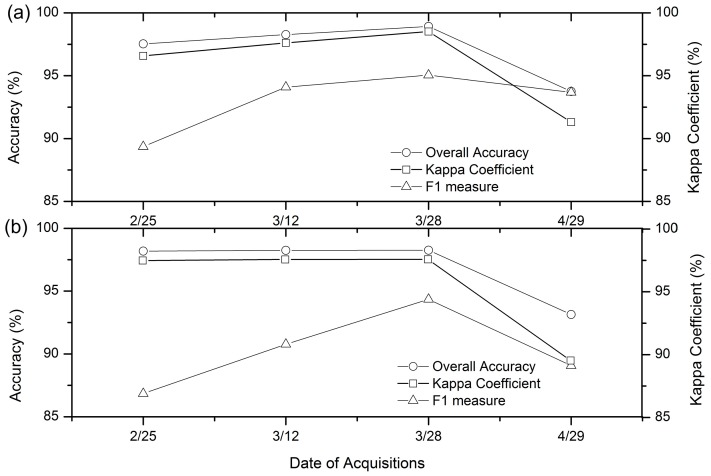
Accuracy of winter wheat using each optical image alone for RF (**a**) and SVM (**b**).

In order to evaluate the effect of the combination of SAR and optical images on the classification accuracy, the optical images were classified by RF and SVM. The accuracy of winter wheat classification is shown in Figure 6. For RF and SVM classifiers, the classification accuracy was the highest at the end of March 2016 (with the overall accuracy and kappa coefficient equal to more than 95% and 0.9500, respectively). Compared with the highest accuracy at the end of March, the classification accuracy at the end of April was obviously reduced. This might be due to the separability of the two crops, which was high at the end of March, in which rapeseed had begun to flower, while the winter wheat was in the jointing stage. However, the separability was reduced after the florescence of rapeseed at the end of April. At the end of February 2016 (early winter wheat growth), winter wheat had the lowest classification accuracy, the reason being that the wheat had a much lower height and coverage at an early stage [20]. 

#### 4.2.3. Classification Results Using SAR and Optical Images

As mentioned above, winter wheat has the highest separability when the optical image corresponds to the jointing period. Thus, in the present study, we selected optical images on 28 March and SAR data to combine. As shown in Table 4, the classification accuracy was improved when multi-temporal SAR and optical images were combined. The application of RF allowed us to increase the overall accuracy from 98.92% to 99.35% (F1 measure = 98.06%). For winter wheat, the addition of SAR images to the optical images increased the F1 measure by 3.23% for RF, and 1.48% for SVM, respectively; SVM was better than RF in terms of the overall accuracy and kappa coefficient, but the F1 measure was lower than RF. For other areas, the classification accuracies were also improved. Some earlier studies have also shown that the classification accuracy could be improved when SAR and optical images were combined [26,33,57,65]. The reason might be that the optical image contains rich spectral information, SAR data has more space texture information, and the combination of SAR and optical images can make up the defects of two kinds of remote sensing images in crop identification to improve the classification accuracy. The results indicated that SAR data is not only a suitable substitution to optical images of crop classification in urban agriculture regions, but also as a supplemental data source, especially in cloud-prone areas.

In this paper, satisfactory classification accuracy was achieved in urban agricultural regions with fragmented planting parcels, and higher classification could be achieved in areas with less-fragmented parcels. Therefore, the results of this study have good application potential for winter wheat classification in other areas.

#### 4.2.4. Incremental Classification Results Using SAR Data 

**Figure 7:**
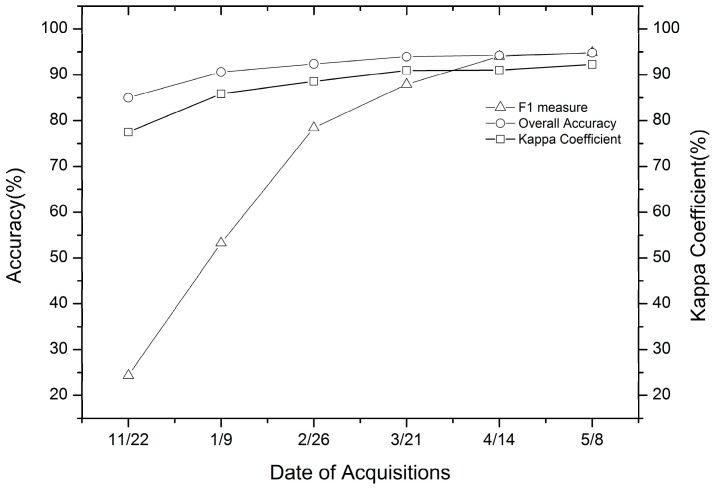
Incremental classification accuracy of winter wheat with backscatter intensity, coherence, and texture images by adding every new image acquisition to all previously available images.

**Figure 8:**
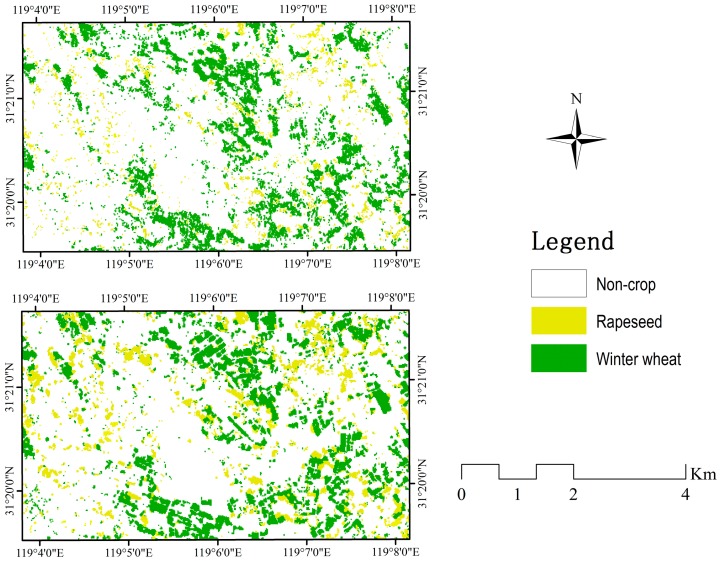
Classification results using the RF classifier. (Top) Using the four SAR datasets (22 November 2015, 9 January 2016, 26 February 2016 and 21 March 2016). (Bottom) Using the combination of S + O.

In order to assess the ability of SAR data to identify winter wheat early, an incremental classification was conducted with backscatter intensity, coherence, and texture images of SAR data. Every new image acquisition was added to all previously available images and was performed using the RF classifier (Figure 7). As shown in Figure 7, the classification accuracy of winter wheat improved as the season progressed. The combination of all six Setinel-1A datasets achieved the highest classification accuracy, using only one Sentinel-1A image with the lowest classification accuracy. This shows that multi-temporal SAR data can provide more useful information to improve classification accuracy [20,66]. In addition, the classification accuracy was satisfactory using four Sentinel-1A images (22 November 2015, 9 January 2016, 26 February 2016 and 21 March 2016) acquired before the jointing period of winter wheat (with an overall accuracy up to 93.91% and a kappa coefficient up to 0.9093, respectively), while the F1 measure was 87.89%. The classification accuracy using four Sentinel-1A datasets is shown in Table 5. As shown in Table 5, for rapeseed fields, the F1 measure was no higher than 75% using the data combination. For others fields, this data combination produced satisfactory accuracy, with both the F1 measure and overall accuracy being higher than 90%. Therefore, using SAR data early in winter wheat, satisfactory classification accuracy can be obtained. Samples of final classification maps are displayed in Figure 8. Inglada et al. [11] evaluated the usefulness of the Sentinel-1A early crop type classification as an optical image supplement and found that a satisfactory land cover map could be obtained early in the season, with a significant improvement in accuracy compared to the use of optical images alone.

## 5. Conclusions

In this paper, the multi-temporal Sentinel-1A data and/or Landsat-8 were classified by RF and SVM classifiers in an urban agriculture region of Southern China. The main conclusions of this paper are: (1) In the urban agriculture region with a complex planting structure, using early- and late-season winter wheat growth multi-temporal Sentinel-1A data for classification, satisfactory classification of winter wheat can be obtained. This indicates that optical data can be replaced by Sentinel-1A images when the effective optical image cannot be obtained due to weather; (2) The classification accuracy of backscatter intensity, alone, was higher than that of the coherence and texture images; (3) The classification accuracy can be effectively improved by coherence and texture information of SAR data, but the overall accuracy of the combination of both features was less than 86%; (4) The result of the combination of SAR and optical data was the best, with the highest overall accuracy of 99.81%, which was better than using optical data alone. For winter wheat, the addition of SAR images to the optical images increased the F1 measure by up to 3.23%; (5) The SVM classifier outperformed the RF slightly in terms of overall accuracy and kappa coefficient, and SVM was much faster than RF; however, RF performed better than SVM in terms of the F1 measure.

As an important component of the Chinese High-Resolution Earth Observation System, the GaoFen-3 (GF-3) was successfully launched in August 2016. GF-3 is China’s first 1-m resolution C-band synthetic aperture radar (SAR) satellite, and has 12 imaging modes. The reported results of this study are also important for the application of GF-3 images in agricultural applications.

## Figures and Tables

**Table 1:** Main characteristics of Sentinel-1A images used in this study.

Acquisition Date	Product	Imaging Mode	Polarization	Incidence Angle
22 November 2015	SLC	IW	VV/VH	33.8
9 January 2016	SLC	IW	VV/VH	33.9
26 February 2016	SLC	IW	VV/VH	39.0
21 March 2016	SLC	IW	VV/VH	33.8
14 April 2016	SLC	IW	VV/VH	33.8
8 May 2016	SLC	IW	VV/VH	33.9

**Table 2:** Numbers of pixels per class for the training and validation data.

Class	Number of Training Pixels	Number of Validation Pixels
Winter wheat	816	928
Rapeseed	807	930
Forest	1186	1095
Water body	1074	1192
Urban	1253	1325

**Table 3:** Different combinations of SAR variables and optical images for winter wheat classification.

ID	Simple Code in This Study	Descriptions of Inputs
A	VH	All six Sentinel-1A images (VH)
B	VV	All six Sentinel-1A images (VV)
C	VV + VH	Dual polarization (VV + VH) of all six Sentinel-1A images
D	T	Textures of all six Sentinel-1A images
E	C	Coherence values of all six Sentinel-1A images
F	VV + VH + T	Textures and dual polarization (VV + VH) of all six Sentinel-1A images
G	VV + VH + C	Coherence values and dual polarization (VV + VH) of all six Sentinel-1A images
H	C + T	Textures and coherence values of all six Sentinel-1A images
I	VV + VH + C + T	Combination of dual polarization (VV + VH), textures, and coherence values of all six Sentinel-1A images
J	S + O	Combination of all six Sentinel-1A images (dual polarization (VV + VH), textures, and coherence values) and Landsat-8 image on 28 March 2016

Notes: VH means VH polarization; VV means VV polarization; C means coherence values; T means textures; S means SAR data; O means optical data.

**Table 4:** Comparison of overall accuracy, kappa coefficient, and F1 measure for each land cover class.

		F1 Measure (%)					Overall Accuracy (%)	Kappa
Classifier	ID	Wheat	Rapeseed	Forest	Urban	Water		
RF	a	50.00	61.80	69.80	80.42	95.58	82.26	0.7351
	b	74.48	64.47	87.25	88.70	96.99	90.10	0.8525
	c	85.72	78.02	87.09	90.24	97.21	91.45	0.8729
	d	15.62	20.26	54.59	82.62	72.62	72.27	0.5741
	e	44.36	21.49	30.76	83.41	67.86	67.27	0.5086
	f	90.34	66.34	87.75	92.42	97.21	92.38	0.8865
	g	92.35	83.20	93.34	95.20	97.26	94.94	0.9263
	h	20.95	26.87	52.67	86.37	74.58	73.82	0.6046
	i	94.83	72.26	91.29	95.27	97.94	94.78	0.9224
	j	98.06	98.85	99.44	99.53	99.25	99.35	0.9905
SVM	a	46.33	54.87	57.71	71.77	95.41	75.47	0.6350
	b	66.36	56.60	80.60	83.42	96.82	86.12	0.7957
	c	83.51	74.20	82.10	88.16	98.09	89.82	0.8490
	d	24.16	39.07	68.49	86.19	85.40	80.59	0.7057
	e	44.14	20.46	41.40	85.10	71.24	69.55	0.5449
	f	91.26	77.92	90.45	94.61	99.20	94.83	0.9231
	g	93.10	84.78	95.51	96.41	97.93	96.19	0.9447
	h	65.65	42.00	74.17	90.62	91.12	85.19	0.7833
	i	93.61	82.08	94.48	96.05	98.06	95.95	0.9399
	j	95.09	96.37	99.31	99.61	99.94	99.81	0.9939

**Table 5:** Accuracy of winter wheat using the four SAR datasets (22 November 2015, 9 January 2016, 26 February 2016 and 21 March 2016).

	Class				
Accuracy Measure	Winter Wheat	Rapeseed	Forest	Urban	Water
F1 measure	87.89	70.75	90.51	94.23	97.83
Overall accuracy/Kappa			93.91/0.9093		
